# NSC777201 exhibits anticancer activity against colorectal cancer cells by inducing multiple types of cell death

**DOI:** 10.1186/s12935-025-04146-6

**Published:** 2025-12-22

**Authors:** Hsin-Hsuan Ho, Sheng-Chieh Wang, Hsu-Shan Huang, Pei-Ming Yang

**Affiliations:** 1 Graduate Institute of Cancer Biology and Drug Discovery, College of Medical Science and Technology, Taipei Medical University, Taipei, 11031 Taiwan; 2 PhD Program for Cancer Molecular Biology and Drug Discovery, College of Medical Science and Technology, Taipei Medical University, Taipei, 11031 Taiwan; 3 TMU Research Center of Cancer Translational Medicine, Taipei, 11031 Taiwan; 4 Cancer Center, Wan Fang Hospital, Taipei Medical University, Taipei, 11696 Taiwan; 5 TMU and Affiliated Hospitals Pancreatic Cancer Groups, Taipei Medical University, Taipei, 11031 Taiwan; 6 PhD Program in Biotechnology Research and Development, College of Pharmacy, Taipei Medical University, 11031 Taipei, Taiwan; 7 School of Pharmacy, National Defense Medical University, 11490 Taipei, Taiwan

**Keywords:** Apoptosis, Autophagy, Colorectal cancer, Ferritinophagy, Ferroptosis

## Abstract

**Background:**

Colorectal cancer (CRC) ranks as the third most prevalent cancer globally, necessitating the development of novel therapeutic strategies. NSC777201, a multi-targeted anticancer agent, has demonstrated efficacy in various cancers, including prostate cancer, non-small cell lung cancer (NSCLC), glioblastoma (GBM), and pancreatic ductal adenocarcinoma (PDAC). However, its potential in CRC remains unexplored.

**Methods:**

In this study, National Cancer Institute (NCI)-60 cancer cell panel screening identified CRC as the most sensitive to NSC777201 treatment. We further validated its in vitro anticancer effects in four CRC cell lines (HCT116, HT29, RKO, and DLD-1) and its in vivo efficacy using HCT116 tumor xenografts in nude mice. Mechanistic studies included analyses of epidermal growth factor receptor (EGFR) and MET protein inhibition and investigations into cell death pathways triggered by NSC777201.

**Results:**

NSC777201 exhibited potent in vitro and in vivo anticancer activities against human CRC cells. NSC777201 inhibited expressions of the EGFR and MET proteins in CRC cells, consistent with findings in other cancers. Mechanistic studies revealed that NSC777201 induced CRC cell death through autophagy, ferroptosis, and ferritinophagy. Moreover, NSC777201-induced ferroptosis was partially dependent on upregulation of 7-dehydrocholesterol (DHC) reductase (DHCR7) and subsequent reduction of the endogenous ferroptosis suppressor, 7-DHC.

**Conclusions:**

These findings highlight NSC777201’s robust anticancer activity in CRC, mediated by multiple cell death pathways, offering promising therapeutic potential for CRC treatment.

## Background

Colorectal cancer (CRC) is a significant global health challenge, ranking among the top three most prevalent cancers and representing the third leading cause of cancer-related mortality in both men and women [1]. Approximately 70% of CRC cases are diagnosed annually at stage II/III [2, 3]. The development of CRC is driven by genetic and epigenetic alterations, notably chromosomal instability (CIN) and microsatellite instability (MSI), with critical mutations frequently occurring in genes such as *APC*, *TP53*, and *KRAS* [4]. Standard treatment approaches for CRC include surgical resection, chemotherapy, and targeted therapies [5, 6]. Chemotherapeutic regimens like FOLFIRI and FOLFOX combine agents such as 5-fluorouracil (5-FU), oxaliplatin, and irinotecan [7], while targeted therapies focus on inhibiting molecular pathways such as the epidermal growth factor receptor (EGFR) and vascular endothelial growth factor (VEGF) pathways [8, 9]. Despite these advancements, the 5-year survival rate for advanced CRC remains relatively low, largely due to the emergence of drug resistance and limited therapeutic options. This highlights the urgent need for continued research to elucidate resistance mechanisms, develop combination therapies, and create novel treatment strategies to improve patient outcomes and survival rates.

Cell death, a biological process resulting in the cessation of cellular function, is essential for removing excess cells during morphogenesis and organogenesis. Although critical for tissue development, maintenance, and repair, cell death can also be induced by injury, illness, or trauma, resulting in pathological outcomes [10, 11]. Cells may undergo either accidental cell death (ACD) or regulated cell death (RCD). RCD involves tightly controlled signaling pathways and molecular mechanisms, such as apoptosis, necroptosis, pyroptosis, ferroptosis, and autophagy-associated cell death [12, 13]. Dysregulation of apoptotic pathways is closely linked to tumorigenesis and various diseases. Malignant cells evade apoptosis by downregulating apoptotic signaling, thereby promoting tumor progression through unchecked proliferation. These aberrations contribute to drug resistance and are a leading cause of clinical treatment failure [14].

NSC777201, also known as compound 7, TC-N19, and GBM-N019 in previous studies [15–19], is a novel anthraquinone derivative initially designed as a topoisomerase inhibitor [15]. Subsequent research demonstrated its broad-spectrum anticancer activity across various cancer types, including prostate cancer, non-small cell lung cancer (NSCLC), pancreatic ductal adenocarcinoma (PDAC), and glioblastoma multiforme (GBM) [15–17, 19, 20]. In NSCLC cells, NSC777201 functions as a dual inhibitor, targeting EGFR and MET protein expressions to overcome resistance to EGFR-tyrosine kinase inhibitors (TKIs) [16]. In GBM cells, its anticancer effects are linked to the downregulation of proteins such as mammalian target of rapamycin (mTOR), signal transducer and activator of transcription 3 (STAT3), and cyclin-dependent kinase 6 (CDK6) [17]. In PDAC cells, a liposomal formulation of NSC777201 (LNSC777201) was developed that demonstrated efficacy through degradation of EGFR/MET proteins [20]. Additionally, inhibition of transmembrane protein 2 (TMEM2) contributes to its anticancer effects in PDAC [19]. Collectively, NSC777201 exhibits multi-targeted anticancer properties.

According to the National Cancer Institute (NCI)−60 screening results, NSC777201 demonstrated notable growth-inhibitory and cytotoxic effects across various cancer cell lines, with particularly strong activity against CRC cells. While previous studies established its anticancer potential in prostate cancer, NSCLC, PDAC, and GBM, its effects on CRC remain unexplored. In this study, we investigated the anticancer activity of NSC777201 in CRC cells, revealing that it exerts significant effects by inducing autophagy, ferroptosis, and ferritinophagy.

## Methods

### Chemicals, reagents, and antibodies

NSC777201 was synthesized as described previously [15]. Oxaliplatin (A10346), 5-fluorouracil (5-FU; A10042), SN-38 (A12011), etoposide (VP-16; A10373), MG132 (A11043), and ferrostatin-1 (A13247) were purchased from AdooQ Bioscience (Irvine, CA, USA). McCoy’s 5 A medium (10–050-CVS) and fetal bovine serum (FBS; 35-010-CV) were purchased from Corning (Corning, NY, USA). Roswell Park Memorial Institute (RPMI)−1640 medium (22400071), L-glutamine (25030-81), non-essential amino acids (NEAAs; 11140050), sodium pyruvate (11360070), an antibiotic-antimycotic solution (PSA contains 10,000 units/mL penicillin, 10,000 µg/mL streptomycin, and 25 µg/mL of amphotericin B; 15240-062), phosphate-buffered saline (PBS) tablets (18912014), radioimmunoprecipitation assay (RIPA) lysis and extraction buffer (89901), and TRIzol reagent (15596026) were purchased from Thermo Fisher Scientific (Waltham, MA, USA). Chloroquine (C6628), Spautin-1 (SML0440), deferoxamine (D9533), dimethyl sulfoxide (DMSO; D5879), phosphatase inhibitor cocktail tablets (04906837001), and protease inhibitor cocktail tablets (11873580001) were purchased from Sigma-Aldrich (Munich, Germany). Z-VAD-FMK (A1902) was purchased from APExBIO (Houston, TX, USA). 3-(4,5-Dimethylthiazol-2-yl)−2,5-diphenyl tetrazolium bromide (MTT; AF-L11939) was purchased from Alfa Aesar (Ward Hill, MA, USA). Anti-solute carrier family 7 member 11 (SLC7A11; ARG57998, 1:1000) and anti-phospholipid hydroperoxide glutathione peroxidase (GPX4; ARG41400, 1:1000) antibodies were purchased from Arigo Biolaboratories (Hsinchu, Taiwan). Anti-phospho-MET (AB5662, 1:500) and anti-caspase-3 (AB13585, 1:300) antibodies were purchased from Abcam (Cambridge, MA, USA). Anti-GAPDH (A19056, 1:20000), anti-vinculin (A2752, 1:2000), anti-ferritin light chain (FTL; A11241, 1:5000), anti-EGFR (4267, 1:1000), anti-phospho-EGFR (2243, 1:1000), and anti-poly(ADP ribose) polymerase (PARP; 9542, 1:1000) antibodies were purchased from Cell Signaling Technology (Danvers, MA, USA). Anti-MET (GTX100637, 1:2500), anti-p62 (GTX100685, 1:1000), anti-SLC3A2/cluster of differentiation 98 (CD98; GTX104108, 1:1000), and Actin (GTX109639) antibodies were purchased from GeneTex (Hsinchu, Taiwan). The anti-light chain 3B (LC3B; 18725-1-AP, 1:2500) antibody was purchased from Proteintech Group (Rosemont, IL, USA). A lipid peroxidation assay kit (E-BC-K880-M) was purchased from Elabscience (Houston, TX, USA). Horseradish peroxidase (HRP)-conjugated anti-rabbit (111-035-003) or anti-mouse (115-035-003) secondary antibodies were purchased from Jackson Laboratory (Bar Harbor, MA, USA). An enhanced chemiluminescence (ECL) reagent (NEL105001EA) was purchased from PerkinElmer (Waltham, MA, USA). The Bradford protein assay (5000006), dual-color protein marker (1610374), 10× sodium dodecyl sulfate (SDS)-glycine running buffer (1610772), Trans-Blot Turbo RTA mini 0.2 μm nitrocellulose transfer kit (1704270), and other reagents for the Western blot analysis were purchased from Bio-Rad Laboratories (Hercules, CA, USA).

**Fig. 1:**
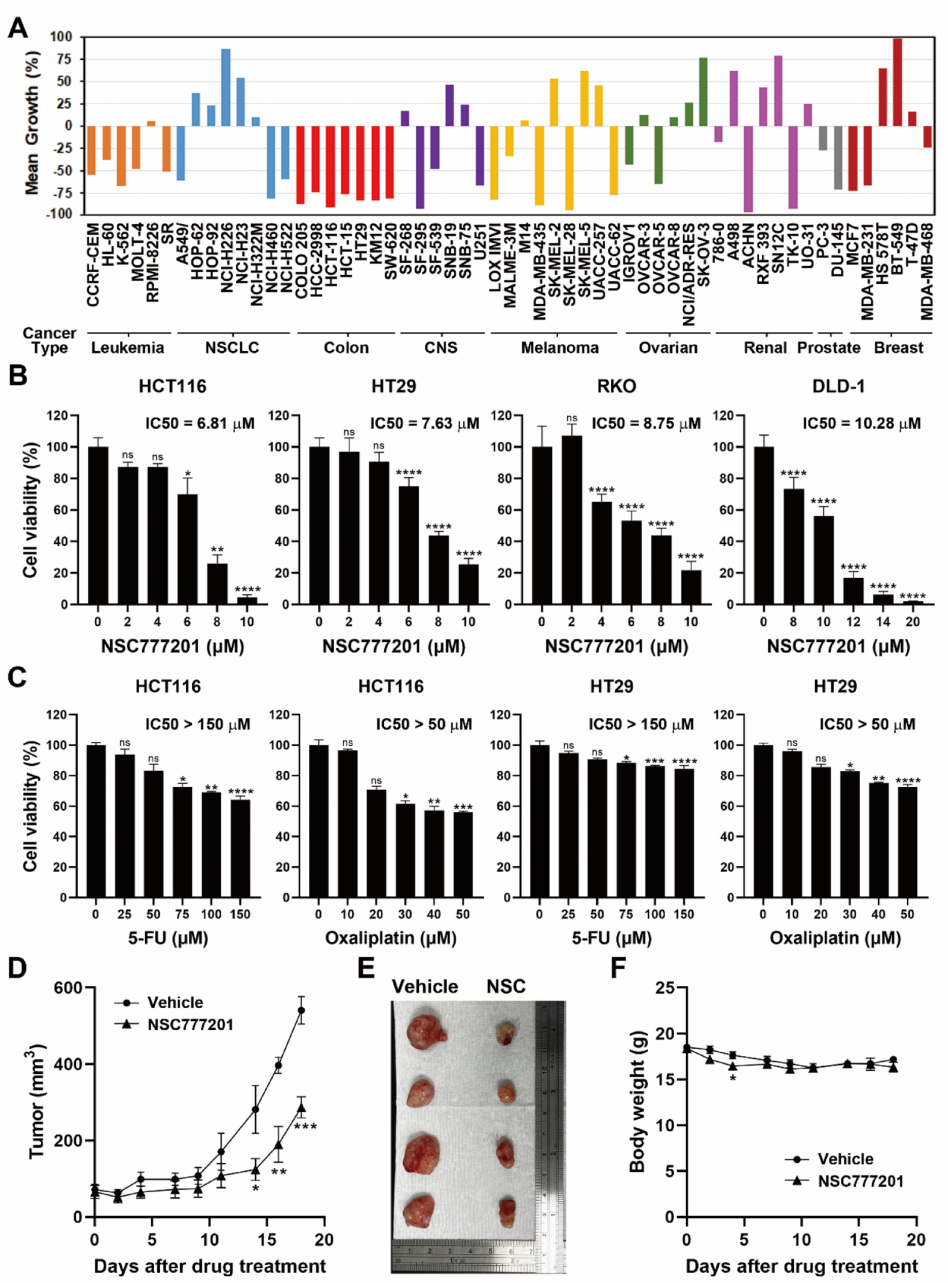
Anticancer activity of NSC777201 in colorectal cancer (CRC) cells. **(A)** NSC777201 was evaluated by the Developmental Therapeutics Program (DTP) at the National Cancer Institute (NCI), part of the National Institutes of Health (NIH), against the NCI-60 cancer cell panel at a single dose of 10 µM. Mean growth percentages are presented. **(B)** HCT116, HT29, RKO, and DLD-1 cells were treated with various concentrations of NSC777201 for 24 h, and cell viability was assessed using an MTT assay. Data are shown as the mean ± SD. Statistical significance is denoted by * *p* < 0.05, ** *p* < 0.01, *** *p* < 0.001, and **** *p* < 0.0001; ns indicates not significant. **(C)** HCT116 and HT29 cells were treated with various concentrations of 5-FU and oxaliplatin for 24 h, and cell viability was assessed using an MTT assay. Data are shown as the mean ± SD. Statistical significance is denoted by * *p* < 0.05, ** *p* < 0.01, *** *p* < 0.001, and **** *p* < 0.0001; ns indicates not significant. **(D-F)** Nude mice bearing HCT116 xenografts were treated with NSC777201 (NSC; 5 mg/kg body weight) or a vehicle control. Tumor volumes (D) and body weights (F) were measured three times per week. Error bars represent the mean ± SEM (*n* = 5). Statistical significance between the vehicle and NSC777201 groups at each time point is indicated by * *p* < 0.05, ** *p* < 0.01, and *** *p* < 0.001. Representative tumor images from treated and control groups are shown in (E)

### National cancer Institute (NCI)−60 screening methodology

NSC777201 was submitted to the Developmental Therapeutics Program (DTP) at the NCI, part of the National Institutes of Health (NIH), for screening against the NCI-60 cancer cell panel at a single dose of 10 µM. The NCI-60 collection comprises 60 human cancer cell lines spanning nine cancer types: leukemia, NSCLC, melanoma, and colon, central nervous system (CNS), ovarian, renal, prostate, and breast cancers. Screening results were presented in a mean graph illustrating the percentage growth of treated cells compared to both the no-drug control and the initial cell count at time zero. This graphical representation facilitates identification of growth inhibition (values between 0 and 100) and lethality (values below 0). A score of 100 indicates no growth inhibition, while a score of 40 signifies 60% growth inhibition. A score of 0 implies no net growth throughout the experiment, and a value of −40 corresponds to 40% lethality. A score of −100 indicates complete cell death [21].

### Cell culture

HCT116 cells were obtained from the Bioresource Collection and Research Center (BCRC; Hsinchu, Taiwan). HT29 cells were kindly provided by Prof. Ya-Wen Cheng (Taipei Medical University, Taipei, Taiwan). RKO and DLD-1 cells were purchased from Horizon Discovery (Cambridge, UK). Cells were cultured in McCoy’s 5 A medium (HCT116) or RPMI-1640 medium (RKO, HT29, and DLD-1) supplemented with 10% FBS, 1% L-glutamine, 1% NEAAs, 1% sodium pyruvate, and 1% PSA, and maintained at 37 °C in a 5% CO_2_ incubator.

### Cell viability assay

Cells (10,000–15,000 cells) were seeded in a 96-well plate. The next day, cells were treated with 100 µL of drug-containing media. After 24 h, 25 µL MTT (2 mg/mL) was directly added to each well, and cells were cultured for an additional 2–4 h. Then, the medium was removed, and MTT formazan was dissolved in 100–200 µL DMSO. Absorbance values at 570 and 650 nm were detected on a microplate reader (Bio-Tek Instruments; Winooski, VT, USA). Cell viability was determined by subtracting the absorbance value at 570 nm from that at 650 nm and normalizing the result to untreated control cells.

### Tumor xenograft model

HCT116 cells (10⁷) were subcutaneously injected into the flanks of nude mice (CAnN.Cg-*Foxn1*^*nu*^/CrlNarl), obtained from the National Laboratory Animal Center (Taipei, Taiwan). Once tumor volumes had reached approximately 50–100 mm³, the mice were randomly assigned to one of two groups: a vehicle group (20 µL DMSO) or the NSC777201 treatment group (5 mg/kg body weight). Treatments were administered via daily intraperitoneal injections, 5 days per week. Tumor volumes and body weights were monitored three times weekly. Tumor volume was calculated using the formula: 0.52×(*a*×*b*^2^), where *a* and *b* respectively represent the tumor’s length and width. The experimental protocol (#SHLAC2024-0034) was reviewed and approved by the Institutional Animal Care and Use Committee at Taipei Medical University.

**Fig. 2:**
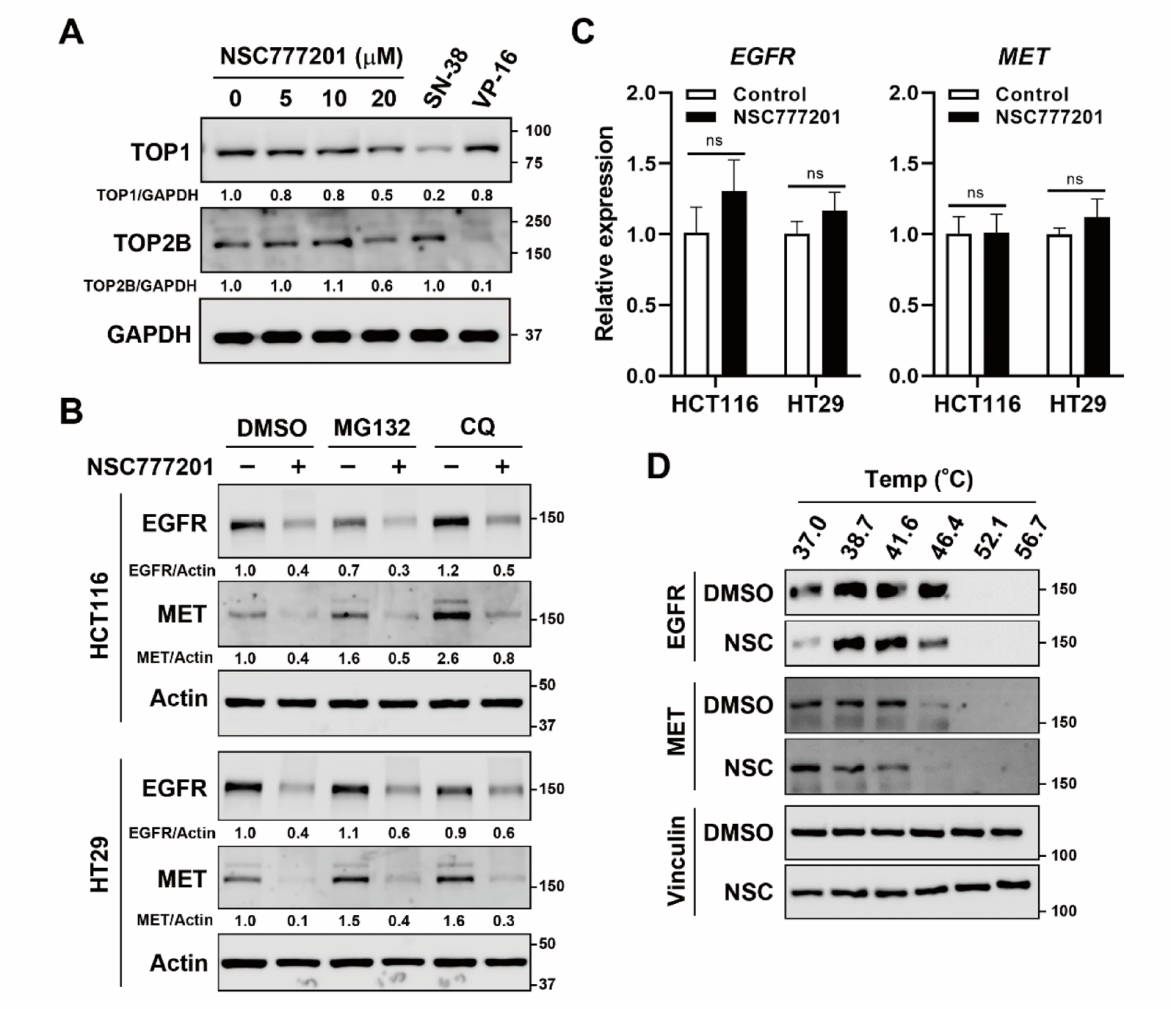
Effect of NSC777201 on its original targets in colorectal cancer (CRC) cells. **(A)** HCT116 cells were treated with 0, 5, 10, or 20 µM NSC777201, 10 µM SN-38, or 200 µM VP-16 for 1 h, followed by a band-depletion assay. **(B)** HCT116 and HT29 cells were pretreated with 10 µM MG132 or 60 µM chloroquine (CQ) for 0.5 h, and then exposed to NSC777201 (6 µM for HCT116 and 8 µM for HT29) for 6 h. Protein expression levels were analyzed by Western blotting. **(C)** HCT116 and HT29 cells were treated with NSC777201 (6 µM for HCT116 and 8 µM for HT29) for 6 h, and mRNA expression levels were analyzed by real-time qPCR. **(D)** HCT116 cells were treated with 6 µM NSC777201 (NSC) for 1 h, followed by a cellular thermal shift assay (CETSA)

### Western blot analysis

Cells were washed twice with ice-cold PBS and centrifuged at 1500 rpm for 5 min. After adding RIPA lysis buffer to the cell pellet, the mixture was vortexed every 5–10 min while kept on ice for 30 min. Samples were then centrifuged at 4 °C at high speed (16,000 ×*g*) for 20 min. The supernatant was collected, and the protein concentration was determined using the Bradford protein assay. Protein lysates (50–100 µg) in loading buffer were loaded into an SDS-polyacrylamide gel electrophoresis (PAGE) gel. After protein separation by electrophoresis, the protein was transferred to a nitrocellulose membrane. The membrane was blocked with 5% non-fat milk for 30 min, followed by incubation with primary antibodies at 4 °C overnight. Subsequently, the membrane was incubated with an HRP-conjugated anti-rabbit or anti-mouse secondary antibody for 2 h. The protein was finally detected using an ECL reagent and visualized with a GE Amersham Imager 600 (GE Healthcare Life Sciences, Marlborough, MA, USA) or an eBlot Touch Imager (eBlot Photoelectric Technology; Shanghai, China).

### Band-depletion assay

A band-depletion assay was employed to assess topoisomerase activity by identifying catalytic topoisomerase-DNA cleavage complexes that were captured by topoisomerase inhibitors. In SDS-PAGE analysis, these topoisomerase-DNA complexes exhibit reduced mobility compared to unbound enzymes [22]. Briefly, cells (5 × 10^6^) were seeded in 6-cm dishes. The next day, cells were treated with 5 mL of the drug-containing medium for 1 h. Then, cells were washed twice with ice-cold PBS and lysed with 1× SDS sample buffer. Protein lysates were collected, vortexed, and then boiled at 100 ℃ for 5 min. Protein levels were analyzed by Western blotting.

### Cellular thermal shift assay (CETSA)

Cells (5 × 10^6^) were seeded in 10-cm dishes. The next day, cells were treated with 8 mL of drug-containing medium for 1 h. Then, cells were trypsinized and washed with PBS. After centrifugation at 1500 rpm for 5 min at room temperature, the pellets were resuspended in 0.5 mL PBS supplemented with 1× of a protease inhibitor cocktail. The suspension was aliquoted to 50 µL per tube and heated from 37 to 62 °C for 3 min in a polymerase chain reaction (PCR) machine. Cells were then immediately lysed by two freeze-thaw cycles. Following centrifugation at 16,000 ×*g* for 15 min at 4 °C, the supernatants were collected and mixed with 6× SDS sample buffer. Proteins were then analyzed by Western blotting.

### Fluorescence microscopy of autophagic vacuoles

Cells were seeded in a µ-Slide eight-well plate (ibidi IB-80826; ibidi; Verona, WI, USA). The next day, cells were treated with 200 µL of drug-containing medium. After 6 h, autophagic vacuole formation was assessed using the Cyto-ID Autophagy Detection Kit (ENZ-51031-K200; Enzo Life Sciences; Farmingdale, NY, USA) according to the manufacturer’s instructions. Briefly, cells were washed twice with PBS containing 5% FBS, and then incubated with Cyto-ID Detection Reagent and Hoechst 33,342. After a 30-min incubation at 37 °C, excess dye was removed by washing, and cells were visualized by fluorescence microscopy.

### Lipid peroxidation assay

Cells were seeded in a µ-Slide eight-well plate. The next day, cells were treated with 200 µL of drug-containing medium. After 6 h, cells were washed twice with PBS, stained with 200 µΜ lipid peroxidation probe (BDP 581/591 C11), and then incubated at 37 °C for 30 min. After staining, cells were washed once with PBS. Green (Ex/Em = 488/510–550 nm) and red (Ex/Em = 561/600–630 nm) fluorescence levels were observed under a fluorescent microscope. The BDP 581/591 C11 probe reacts with lipid radicals formed during lipid peroxidation, resulting in a fluorescence color change from red to green, indicating the occurrence of lipid peroxidation.

**Fig. 3:**
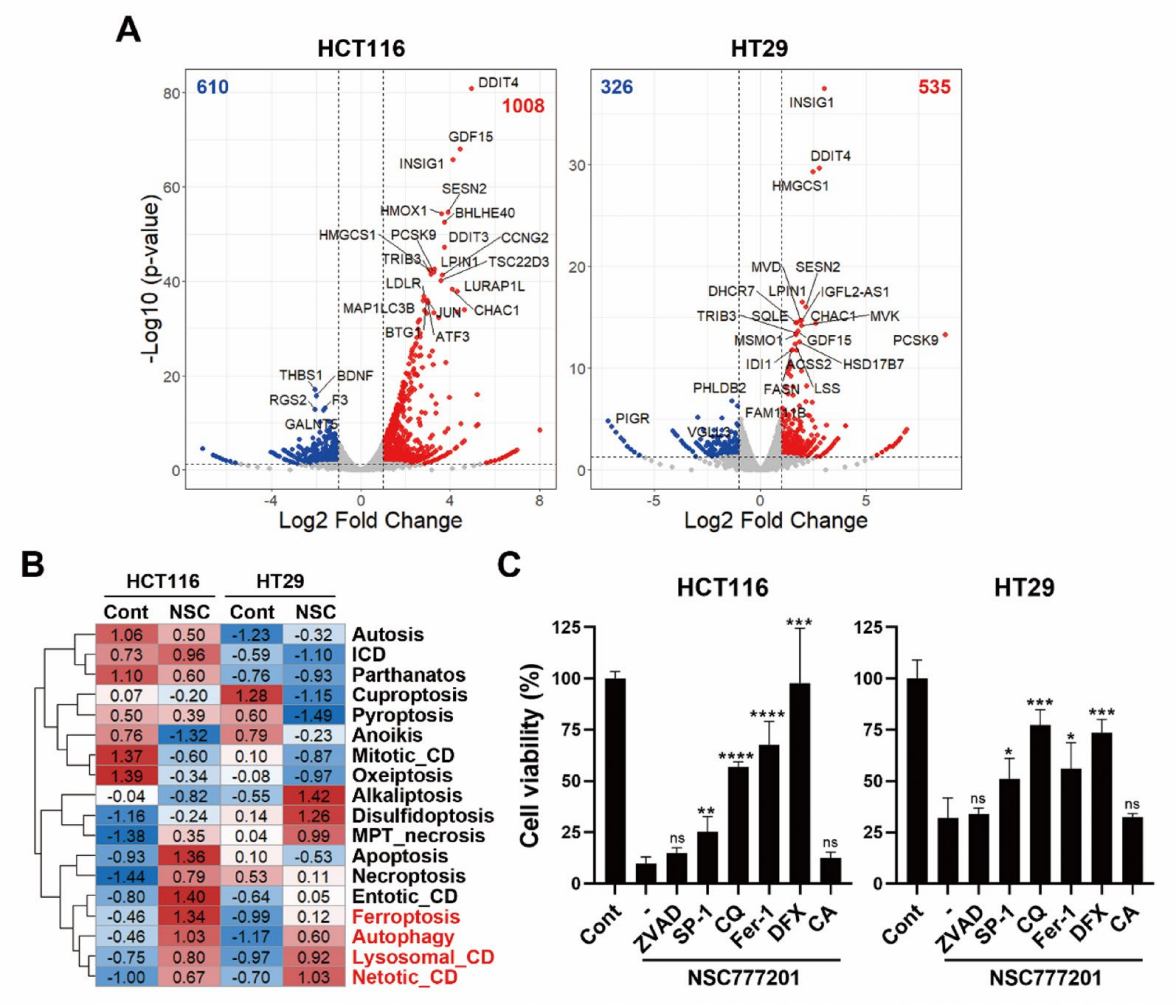
Prediction of NSC777201-induced cell death in colorectal cancer (CRC) cells using an RNA sequencing (RNA-Seq) analysis. **(A)** HCT116 and HT29 cells were treated with NSC777201 (6 µM for HCT116 and 8 µM for HT29) for 6 h, followed by an RNA-Seq analysis. Differential gene expression was visualized using volcano plots. **(B)** A gene set variation analysis (GSVA) was conducted against regulated cell death (RCD) gene signatures. Those highlighted in red indicate potential RCD pathways induced by NSC777201. **(C)** HCT116 and HT29 cells were treated with 8 µM NSC777201 for 24 h, with or without 100 µM Z-VAD-FMK (ZVAD), 1 µM spautin-1 (SP-1), 20 µM chloroquine (CQ), 1 µM ferrostatin-1 (Fer-1), 10 µM deferoxamine (DFX), or 50 µM CA-074Me (CA). Cell viability was assessed using an MTT assay and normalized to the respective controls treated with the inhibitors alone. Data are presented as the mean ± SD. * *p* < 0.05, ** *p* < 0.01, *** *p* < 0.001; ns, not significant

### Fluorescence imaging of intracellular ferrous iron by ferroorange staining

Cells were seeded in a µ-Slide eight-well plate. The next day, cells were treated with 200 µL of the drug-containing medium. After 6 h, intracellular ferrous iron levels were examined using the FerroOrange probe (F374; Dojindo Laboratories; Kumamoto, Japan) according to the manufacturer’s instructions. Briefly, cells were washed three times with serum-free medium and then incubated with 1 µΜ FerroOrange working solution. After a 30-min incubation at 37 °C, cells were observed under a fluorescence microscope.

### RNA sequencing (RNA-Seq) analysis

HCT116 and HT29 cells were respectively treated with 6 and 8 µM NSC777201 for 6 h. Total RNA was extracted using the TRIzol reagent and subjected to an RNA-Seq analysis (one biological replicate with two technical replicates) by AllBio Life (Taichung City, Taiwan). Sequencing was performed on the Illumina NovaSeq platform using a 2 × 150-bp paired-end configuration. Gene expression was quantified using HTSeq (v0.6.1), and differentially expressed genes (DEGs) were identified using the Bioconductor package EdgeR (v3.28.1) based on the criteria of |fold-change| > 2 and *p* < 0.05.

### Real-time quantitative (q)PCR

Total RNA were reverse-transcribed into complementary (c)DNA using an iScript cDNA Synthesis Kit (#1708891) purchased from Bio-Rad Laboratories. The qPCR was carried out on a Quantstudio 1 Real-Time PCR System (Applied Biosystems, Foster City, CA, USA) utilizing the IQ2 SYBR Green Fast qPCR System Master Mix (#DBU-006; Bio-Genesis Technologies, Taipei, Taiwan). The following primer pairs were used: 5’-TTCCTCCCAGTGCCTGAAT-3’ (forward) and 5’-GGTTCAGAGGCTGATTGTGAT-3’ (reverse) for human EGFR; 5’-AGTCATAGGAAGAGGGCATT-3’ (forward) and 5’-CTTCACTTCGCAGGCAGA-3’ (reverse) for human MET; 5’-GCTGCAAAATCGCAACCCAA-3’ (forward) and 5’-GCTCGCCAGTGAAAACCAGT-3’ (reverse) for human DHCR7; 5’-GGAAGACCAGCAAGGAGGAA-3’ (forward) and 5’-ACTGCACGGCCAAGTCAATA-3’ (reverse) for human farnesyl-diphosphate farnesyltransferase 1 (FDFT1); 5’-CTGACCTTTATGATGATGCAGC-3’ (forward) and 5’-CAGGCTTTTCTTAGTTGATGCA-3’ (reverse) for human squalene monooxygenase (SQLE); and 5’-GTTGCTATCCAGGCTGTGCT-3’ (forward) and 5’-AGGGCATACCCCTCGTAGAT-3’ (reverse) for human β-Actin (ACTB). Relative expression levels were determined by the comparative cycle threshold (ΔCT) method.

**Fig. 4:**
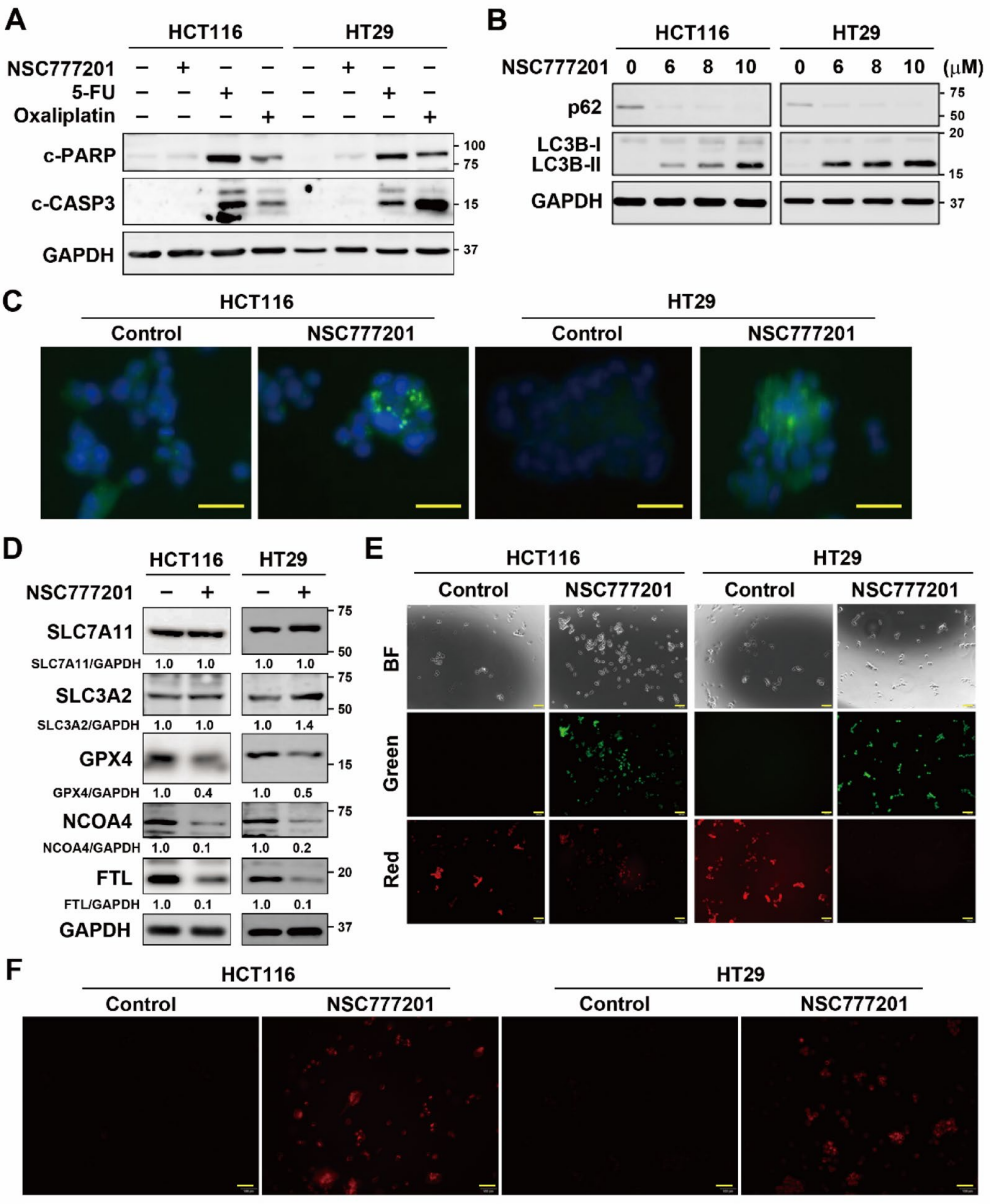
Validation of NSC777201-induced cell death in colorectal cancer (CRC) cells. **(A)** HCT116 and HT29 cells were treated with NSC777201 (6 µM for HCT116 and 8 µM for HT29), 150 µM 5-FU, or 50 µM oxaliplatin for 24 h. Protein expression levels were analyzed by Western blotting. **(B)** HCT116 and HT29 cells were treated with 6, 8, or 10 µM of NSC777201 for 6 h. Protein expression levels were assessed by Western blotting. **(C)** HCT116 and HT29 cells were treated with NSC777201 (6 µM for HCT116 and 8 µM for HT29) for 6 h, and then stained with Cyto-ID Autophagy Detection Reagent. The Cyto-ID fluorescence was observed under a fluorescence microscope. Scale bar = 50 μm. **(D)** HCT116 and HT29 cells were treated with NSC777201 (6 µM for HCT116 and 8 µM for HT29) for 6 h. Protein expression levels were analyzed by Western blotting. **(E)** HCT116 and HT29 cells were treated with NSC777201 (6 µM for HCT116 and 8 µM for HT29) for 6 h, and a lipid peroxidation assay was performed. Scale bar = 100 μm. **(F)** HCT116 and HT29 cells were treated with NSC777201 (6 µM for HCT116 and 8 µM for HT29) for 6 h, and then stained with the FerroOrange probe. Fluorescence was observed under a fluorescence microscope. Scale bar = 100 μm

### 7-Dehydrocholesterol (7-DHC) enzyme-linked immunosorbent assay (ELISA)

HCT116 and HT29 cells were respectively treated with 6 and 8 µM NSC777201 for 6 h. Cells were lysed using RIPA buffer and further diluted with PBS to detect intracellular 7-DHC levels using an ELISA kit (CED057Hu; Cloud-Clone, Houston, TX, USA), following the manufacturer’s instructions. Data were normalized to the relative protein content.

### Statistical analysis

All experiments were performed with at least three independent biological replicates. Quantitative results are presented as the mean ± standard deviation (SD) or mean ± standard error of the mean (SEM), as indicated. Statistical analyses were carried out with GraphPad Prism 9 (GraphPad Software, San Diego, CA, USA). For comparisons between two groups, an unpaired two-tailed Student’s *t*-test was used; for multiple-group comparisons, a one-way analysis of variance (ANOVA) followed by Tukey’s post-hoc test was applied. *p* < 0.05 was considered statistically significant.

## Results

### NSC777201 exhibits in vitro and in vivo anticancer activities in CRC cells

NCI-60 screening results (Table 1; Fig. 1A) revealed that NSC777201 exerted significant growth-inhibitory and cytotoxic effects on cancer cells, with particularly strong activity against CRC cells. While previous studies demonstrated the anticancer efficacy of NSC777201 in prostate cancer, NSCLC, PDAC, and GBM [15–17, 19, 20], its activity against CRC cells remains unexamined. To investigate this, four CRC cell lines (HCT116, HT29, RKO, and DLD-1) were treated with NSC777201 for 24 h, and cell viability was assessed using an MTT assay. As shown in Fig. 1B, NSC777201 significantly reduced cell viability in all four cell lines, with 50% inhibitory concentration (IC_50_) values of 6.81, 7.63, 8.75, and 10.28 µM for HCT116, HT29, RKO, and DLD-1 cells, respectively. Compared to its effects on other cancer cell lines, NSC777201 displayed superior potency in inhibiting CRC cell proliferation (Table 2). Furthermore, NSC777201 demonstrated superior anticancer activity compared to standard treatments with 5‑FU and oxaliplatin (Fig. 1C). The in vivo anticancer activity of NSC777201 was further confirmed using a nude mouse model bearing HCT116 tumor xenografts. Mice were intraperitoneally administered NSC777201 (5 mg/kg body weight), and tumor growth was monitored. As shown in Fig. 1D and E, NSC777201 markedly suppressed the growth rate of HCT116 tumors. Although a slight reduction in body weight was observed during the early stages of treatment, the mice subsequently recovered (Fig. 1F). In addition, neither the appearance nor physical activity was significantly impacted by NSC777201 treatment (laboratory observations), suggesting that NSC777201 was well-tolerated and caused no or few severe adverse effects. Together, these findings demonstrated that NSC777201 possesses potent in vitro and in vivo anticancer activities against human CRC cells.

**Table 1:** Quantification of growth-inhibitory and lethal effects of NSC777201 in the NCI-60 screening

Cancer Type	Growth Inhibition	Lethality
Leukemia NSCLC CRC CNS cancer Melanoma Ovarian cancer Renal cancer Prostate cancer Breast cancer	1/6 (16.7%) 5/8 (62.5%) 0/7 (0%) 3/6 (50%) 4/9 (44.4%) 4/6 (66.7%) 5/7 (71.4%) 0/2 (0%) 3/6 (50%)	5/6 (83.3%) 3/8 (37.5%) 7/7 (100%) 3/6 (50%) 5/9 (55.6%) 2/6 (33.3%) 2/7 (28.6%) 2/2 (100%) 3/6 (50%)
	25/57 (43.9%)	32/57 (56.1%)

NSCLC, non-small cell lung cancer; CRC, colorectal cancer; CNS, central nervous system

**Table 2:** Comparisons of the 50% inhibitory concentration (IC_50_) values and treatment times of NSC777201 across different cancer cell types

Cancer type/Cell line	IC_50_ (µM)	Time	Reference
Prostate cancer PC-3 DU145	5.01 2.84	72 h	[15]
NSCLC PC9 PC9GR CL97	4.9 5.7 5.5	24 h	[16]
PDAC AsPC-1 PANC-1 SUIT-2 PANC-1	4.69 4.78 11 16	72 h 48 h	[20] [19]
GBM U251 U87MG	6 ~ 8 2 ~ 4	48 h	[17]
CRC HCT116 HT29 RKO DLD-1	6.81 7.63 8.75 10.28	24 h	This study

NSCLC, non-small cell lung cancer; PDAC, prostate ductal adenocarcinoma; GBM, glioblastoma multiforme; CRC, colorectal cancer

### NSC777201 inhibits protein expressions of EGFR and MET in CRC cells

NSC777201 was initially synthesized as a topoisomerase inhibitor [15]. To determine whether its anticancer effects in CRC cells are linked to topoisomerase inhibition, we performed a band-depletion assay to assess topoisomerase activity. This assay detects catalytic topoisomerase-DNA cleavage complexes stabilized by topoisomerase inhibitors, which exhibit reduced mobility in SDS-PAGE compared to unbound enzymes [23, 24]. As shown in Fig. 2A, the topoisomerase 1 (TOP1) inhibitor, SN-38, and the topoisomerase 2 (TOP2) inhibitor, VP-16, significantly reduced levels of TOP1 and TOP2B, respectively. In contrast, NSC777201 caused only minimal reductions in TOP1 and TOP2B levels, even at a high concentration (20 µM). These findings suggest that although NSC777201 exhibits some topoisomerase inhibitory activity, this mechanism alone does not fully account for its anticancer effects in CRC cells.

Our previous study showed that NSC777201 promotes degradation of both EGFR and MET via the ubiquitin-proteasome pathway [16]. To determine whether NSC777201 similarly regulates EGFR and MET in CRC cells, we treated HCT116 and HT29 cells with NSC777201 and measured protein and mRNA levels. As expected, NSC777201 reduced EGFR and MET protein levels without affecting their mRNA expression in both cell lines (Fig. 2B and C). However, treatment with MG132, a proteasome inhibitor, failed to restore EGFR and MET protein levels (Fig. 2B), suggesting that the ubiquitin-proteasome pathway may not mediate NSC777201‑induced degradation of EGFR and MET. Since lysosomal degradation is another major proteolytic route [25], we next tested chloroquine, a lysosome inhibitor, and found that it likewise did not rescue EGFR or MET protein levels (Fig. 2B).

**Fig. 5:**
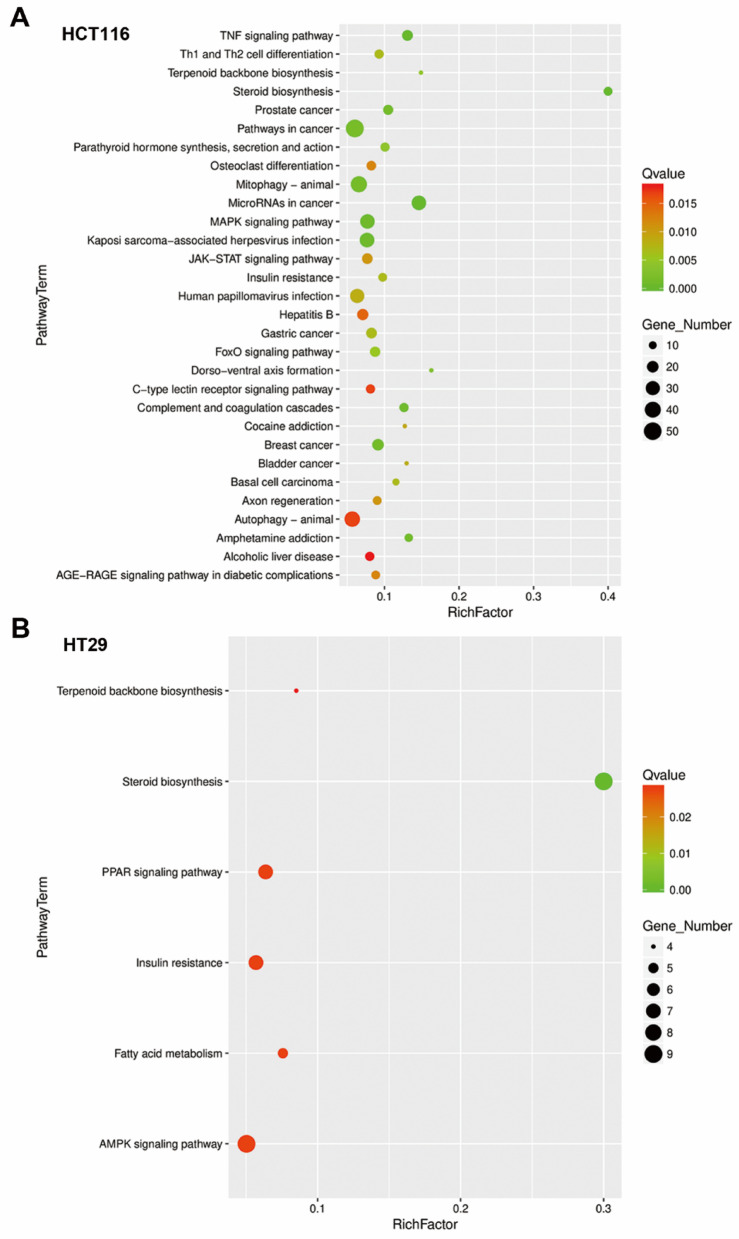
Kyoto Encyclopedia of Genes and Genomes (KEGG) pathway enrichment analysis of NSC777201-treated colorectal cancer (CRC) cells. **(A**,** B)** KEGG pathway enrichment analysis was performed on differentially expressed genes (DEGs) in NSC777201-treated HCT116 (**A**) and HT29 (**B**) cells

To determine whether NSC777201 directly interacts with EGFR and/or MET, we conducted a cellular thermal shift assay (CETSA). A CETSA assesses drug-target engagement in intact cells by measuring the increasing thermal stability of target proteins upon drug binding [26]. NSC777201 treatment slightly reduced the thermal stability of EGFR and MET in HCT116 cells (Fig. 2C). In summary, these findings indicated that NSC777201 exerts its anticancer activity in CRC cells through suppression of EGFR and MET protein expressions. However, the exact mechanism warrants further investigation.

### NSC777201 induces multiple types of cell death in CRC cells

To elucidate the mechanisms by which NSC777201 kills CRC cells, we employed bioinformatics approaches. RNA-Seq analysis was performed on NSC777201-treated HCT116 and HT29 cells, to identify DEGs (Fig. 3A). In HCT116 cells, 610 genes were downregulated, and 1008 genes were upregulated. In HT29 cells, 326 genes were downregulated, and 535 genes were upregulated. A gene set variation analysis (GSVA [27]) was conducted using gene signatures associated with RCD. We hypothesized that if NSC777201 induces specific types of cell death, the corresponding gene signatures would show altered expressions. As shown in Fig. 3B, NSC777201 upregulated the gene signatures for ferroptosis, autophagy, lysosomal cell death, and netotic cell death. Given that NETosis is a distinct form of cell death primarily occurring in neutrophils [28], we focused on ferroptosis, autophagy, and lysosomal cell death. Co-treatment experiments were performed with inhibitors targeting ferroptosis (ferrostatin-1 (Fer-1) and deferoxamine (DFX)), autophagy (Spautin-1 (SP-1) and chloroquine (CQ)), and lysosomal cell death (DFX and CA-074Me (CA)). NSC777201-induced cytotoxicity was rescued by DFX, Fer-1, CQ, and SP-1 (Fig. 3C), suggesting that NSC777201 induces ferroptosis and autophagy. As a negative control, the apoptosis inhibitor, Z-VAD-FMK (ZVAD), was included, but it failed to rescue cells from NSC777201-induced cell death (Fig. 3C).

**Fig. 6:**
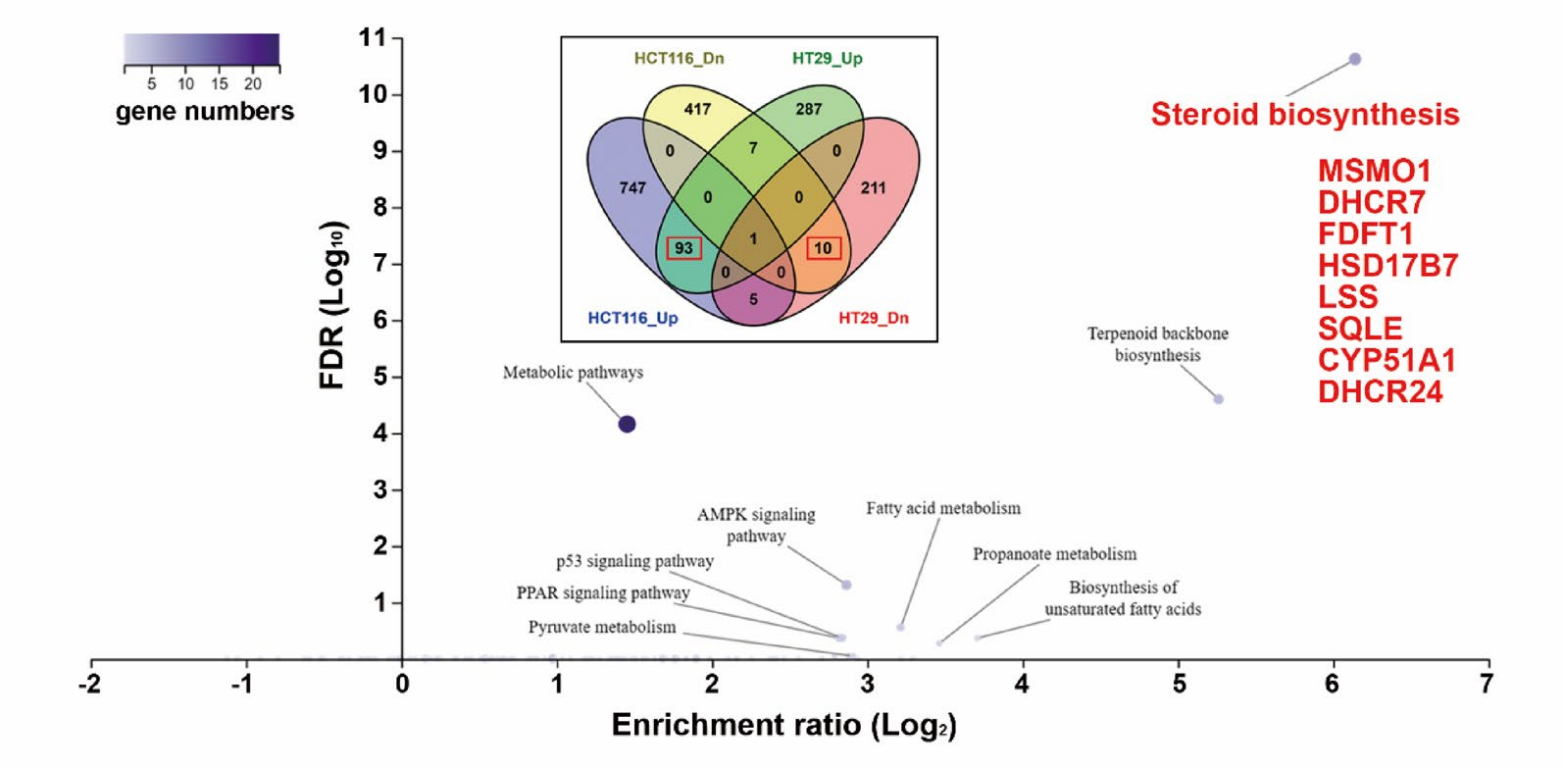
Kyoto Encyclopedia of Genes and Genomes (KEGG) pathway enrichment analysis for common genes in NSC777201-treated colorectal cancer (CRC) cells. Overlapping differentially expressed genes (DEGs, as shown in the embedded figure) in NSC777201-treated HCT116 and HT29 cells were subjected to a KEGG pathway enrichment analysis using the WebGestalt online tool

To validate these findings, markers for these RCD pathways were analyzed via Western blotting. To confirm that NSC777201 does not induce apoptosis, cleavage of PARP and caspase-3 was examined, with 5-FU and oxaliplatin serving as positive controls [29]. Unlike 5-FU and oxaliplatin, NSC777201 did not induce PARP or caspase-3 cleavage (Fig. 4A). Autophagy was assessed by analyzing the conversion of LC3B-I to LC3B-II and the degradation of p62. NSC777201 was found to induce LC3B-II accumulation and p62 degradation (Fig. 4B). In addition, NSC777201 induced the formation of autophagic vacuoles (Fig. 4C), further confirming the induction of autophagy by NSC777201. For ferroptosis, three key repressors (SLC7A11, SLC3A2, and GPX4) were examined. NSC777201 inhibited GPX4 expression (Fig. 4D). Ferritinophagy, a selective autophagic process where ferritin is degraded to release iron, was also evaluated. This process is mediated by NCOA4, which binds ferritin heavy (FTH) and light chains (FTL), targeting them for lysosomal degradation [30]. NSC777201 induced NCOA4 and FTL degradation, indicating activation of ferritinophagy. Given the significant contribution of ferroptosis to NSC777201-induced cytotoxicity (Fig. 3C), lipid peroxidation and intracellular ferrous iron assays were conducted for further validation. Fluorescence shifts from red to green confirmed the induction of lipid peroxidation by NSC777201 (Fig. 4E). Moreover, NSC777201 also upregulated intracellular ferrous iron levels (Fig. 4F). In conclusion, NSC777201 induced both autophagy and ferroptosis in CRC cells, contributing to its cytotoxic effects.

**Fig. 7:**
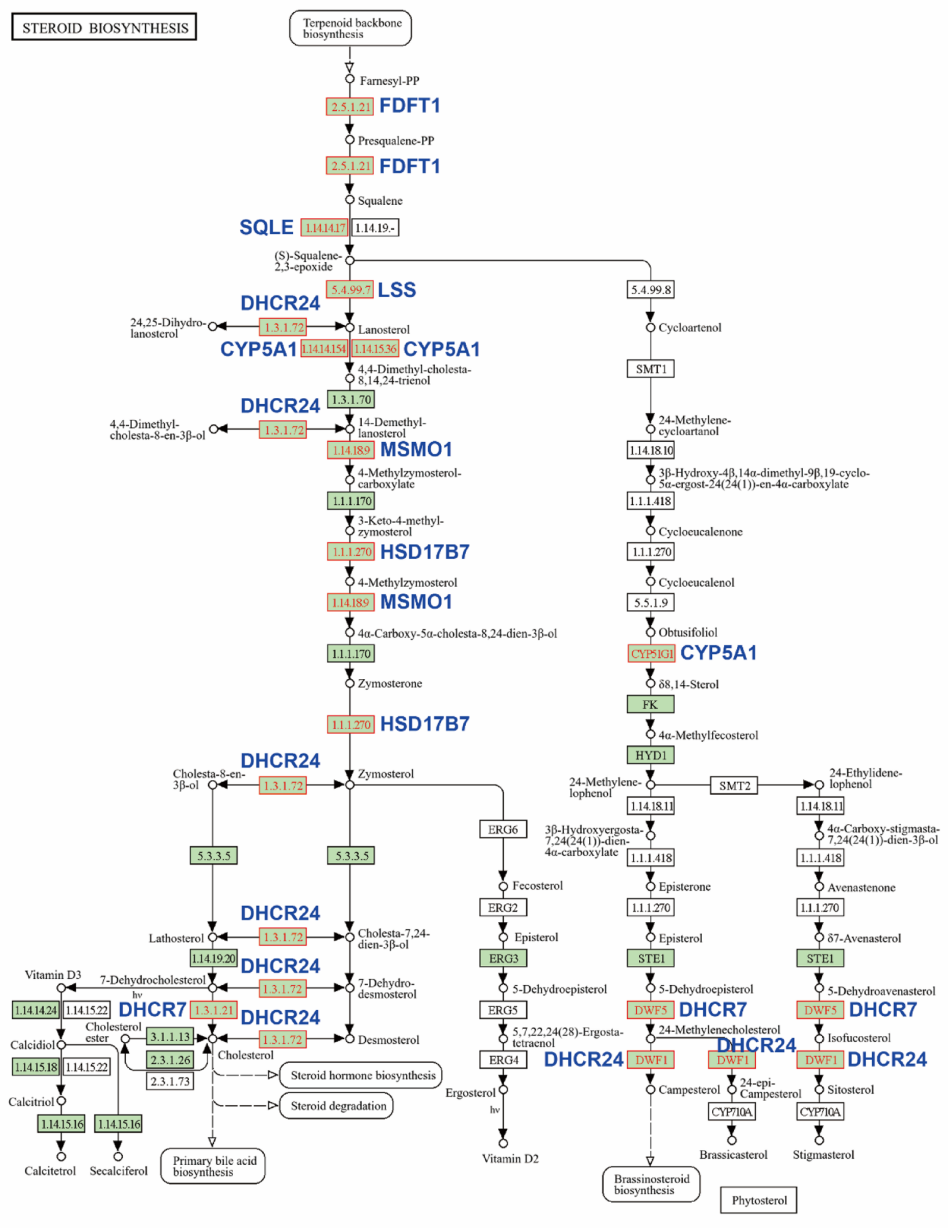
NSC777201 upregulates genes related to cholesterol biosynthesis in colorectal cancer (CRC) cells. Pathway mapping of steroid biosynthesis pathways. Genes responsible for enrichment are highlighted in blue

### RNA-Seq analysis reveals the potential mechanism for the anticancer activity of NSC777201

To investigate potential mechanisms underlying the anticancer activity of NSC777201 in CRC cells, we analyzed RNA-Seq data from NSC777201-treated HCT116 and HT29 cells. DEGs in HCT116 and HT29 cells were independently subjected to a Kyoto Encyclopedia of Genes and Genomes (KEGG [31]) pathway enrichment analysis. As shown in Fig. 5, the most significantly enriched and common pathway in both cell lines was “steroid biosynthesis.” To confirm that “steroid biosynthesis” is the most critical pathway affected by NSC777201, the 93 commonly upregulated and 10 commonly downregulated genes in both cell lines were subjected to a pathway enrichment analysis using the online WebGestalt tool [32]. Consistently, “steroid biosynthesis” was significantly enriched in the 93 commonly upregulated genes (Fig. 6). The key genes responsible for this enrichment included farnesyl-diphosphate farnesyltransferase 1 (*FDFT1*), squalene epoxidase (*SQLE*), lanosterol synthase (*LSS*), cytochrome P450 family 51 subfamily A member 1 (*CYP51A1*), methylsterol monooxygenase 1 (*MSMO1*), hydroxysteroid 17-beta dehydrogenase 7 (*HSD17B7*), 24-dehydrocholesterol reductase (*DHCR24*), and 7-dehydrocholesterol reductase (*DHCR7*) (Fig. 6). KEGG pathway mapping of these genes revealed that NSC777201 upregulated expressions of several key enzymes involved in cholesterol biosynthesis (Fig. 7).

**Fig. 8:**
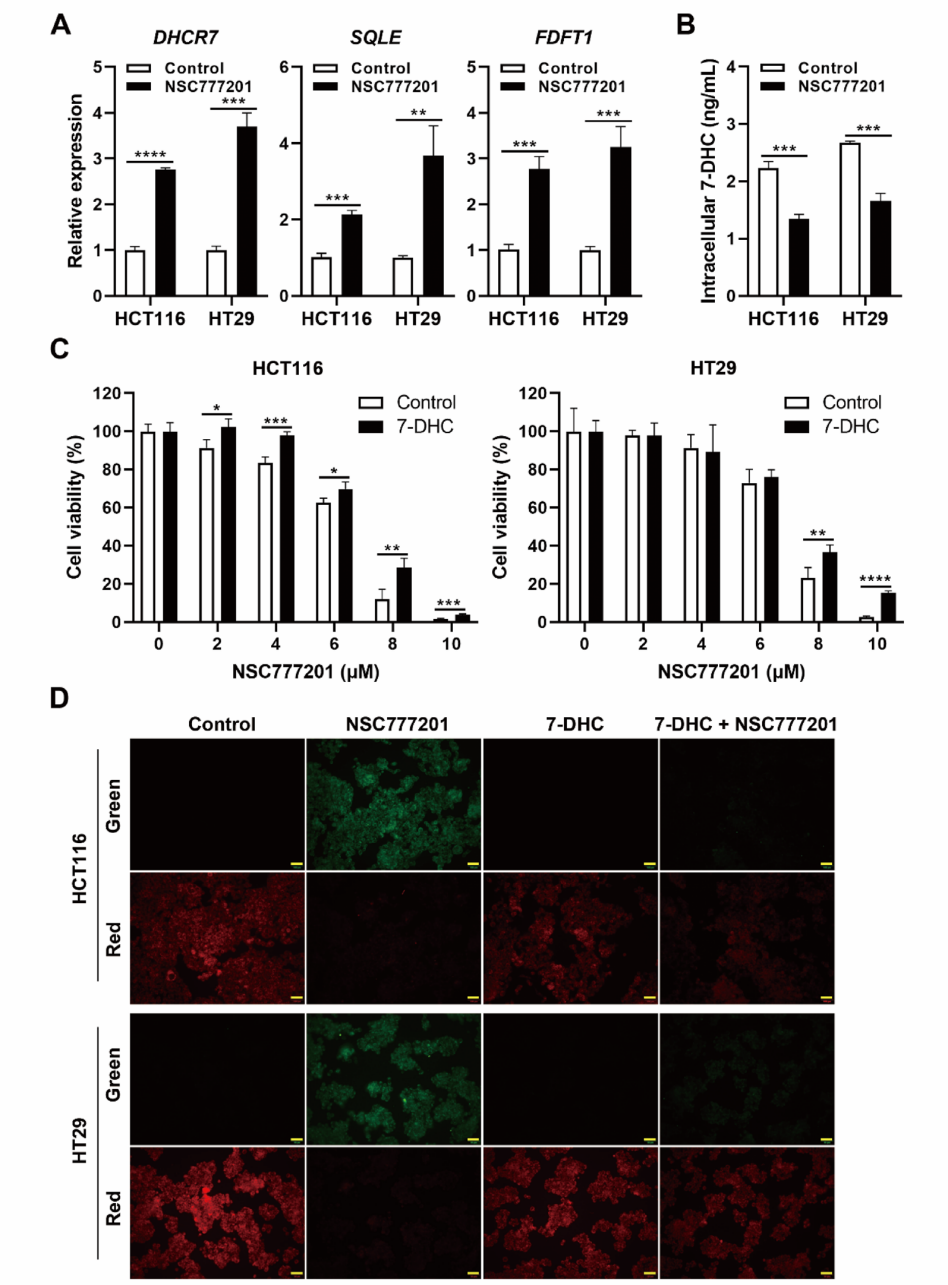
Impact of exogenous 7-dehydrocholesterol (7-DHC) on NSC777201-induced cytotoxicity and lipid peroxidation. **(A)** HCT116 and HT29 cells were treated with NSC777201 (6 µM for HCT116 and 8 µM for HT29) for 6 h, and a real-time qPCR was performed to measure gene expressions. ** *p* < 0.01, *** *p* < 0.001, **** *p* < 0.0001. **(B)** HCT116 and HT29 cells were treated with NSC777201 (6 µM for HCT116 and 8 µM for HT29) for 6 h, and an ELISA was performed to measure intracellular 7-DHC levels. *** *p* < 0.001. **(C)** HCT116 and HT29 cells were treated with indicated doses of NSC777201 for 24 h, with or without 30 µM 7-DHC. * *p* < 0.05, ** *p* < 0.01, *** *p* < 0.001, **** *p* < 0.0001. **(D)** HCT116 and HT29 cells were treated with NSC777201 (6 µM for HCT116 and 8 µM for HT29) with or without 30 µM 7-DHC for 6 h, and a lipid peroxidation assay was performed. Scale bar = 100 μm

To validate the results of the RNA-Seq analysis, three key genes (*DHCR7*, *FDFT1*, and *SQLE*) were analyzed using a qPCR. As shown in Fig. 8A, NSC777201 treatment significantly upregulated these genes. Recent research showed that the cholesterol precursor, 7-DHC, is an effective anti-ferroptosis metabolite. 7-DHC protects cells from phospholipid peroxidation in plasma membranes and mitochondria by redirecting phospholipid peroxidation pathways, thereby mitigating ferroptosis. In the absence of functional DHCR7, 7-DHC accumulates [33, 34]. In this study, RNA-Seq and real-time qPCR analyses revealed that NSC777201 upregulated DHCR7 expression, potentially reducing intracellular 7-DHC levels and thereby inducing ferroptosis. Indeed, NSC777201 significantly reduced intracellular 7-DHC levels (Fig. 8B). In addition, the addition of exogenous 7-DHC restored cell viability and inhibited lipid peroxidation following NSC777201 treatment (Fig. 8C and D). These findings suggest that NSC777201 induces ferroptosis by upregulating DHCR7 and reducing endogenous 7-DHC levels.

### Clinical relevance of DHCR7 in CRC patients

**Fig. 9:**
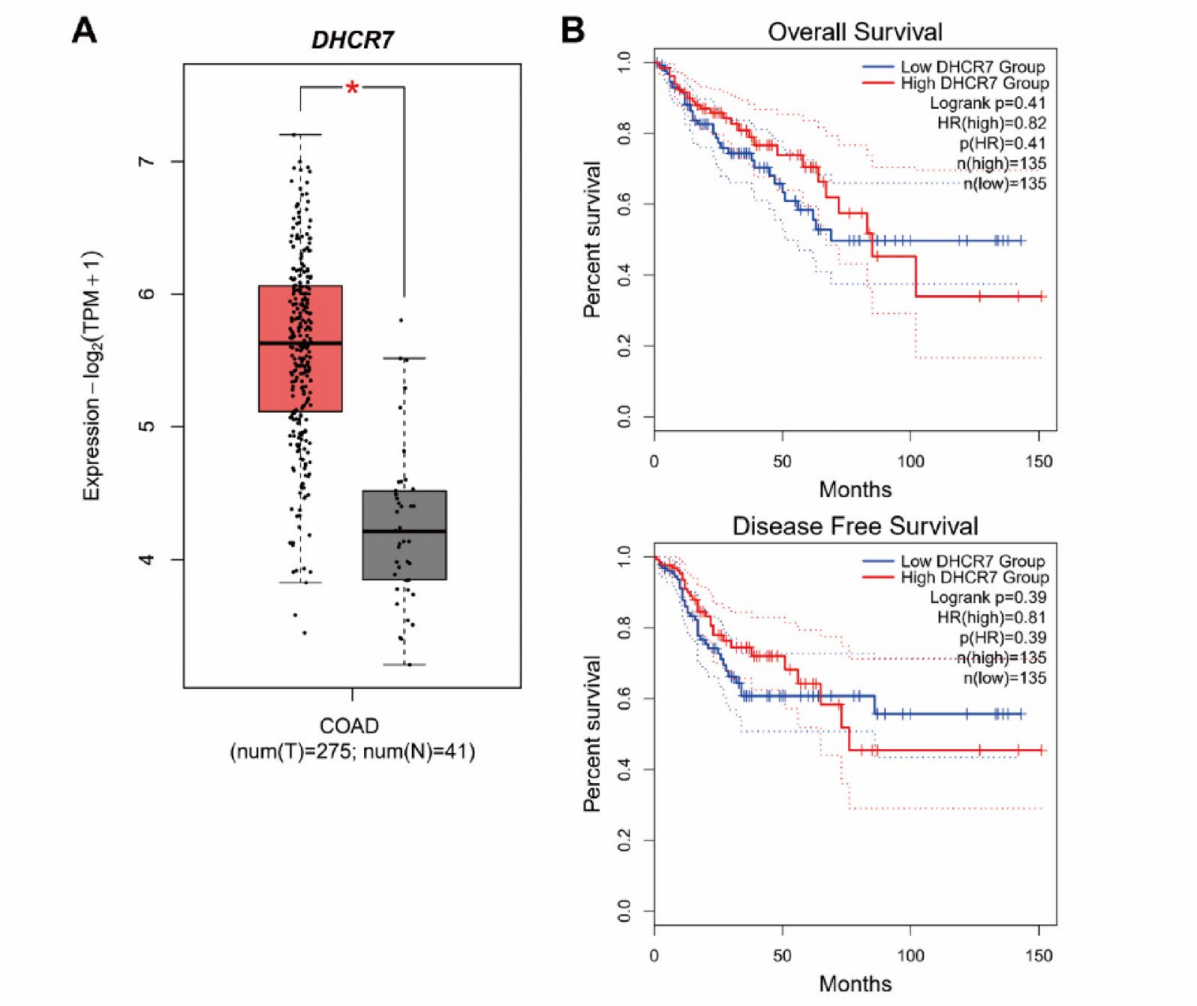
7**-**Dehydrocholesterol reductase (DHCR7) expression and prognostic value in colorectal cancer (CRC) patients. **(A)** Comparison of *DHCR7* mRNA levels in CRC tissues versus adjacent normal tissues, analyzed using the GEPIA2 web tool. **(B)** Kaplan-Meier analysis of overall and disease-free survival according to *DHCR7* mRNA expression in CRC patients, performed using the GEPIA2 web tool

To assess the clinical significance of DHCR7 in CRC, we analyzed patient data from The Cancer Genome Atlas (TCGA) via the GEPIA2 website (http://gepia2.cancer-pku.cn/) [35]. DHCR7 expression was significantly elevated in CRC tissues compared to normal controls (Fig. 9A), but it was not associated with either overall or disease-free survival (Fig. 9B). Because we showed that NSC777201 induces ferroptosis by upregulating DHCR7 and depleting endogenous 7-DHC, CRC patients with high DHCR7 expression may particularly benefit from NSC777201 therapy.

## Discussion

Previous studies in other cancer types demonstrated that the major mechanism of NSC777201’s ability to kill cancer cells is through apoptosis [16, 17, 20]. In our study, however, apoptosis was not the major type of cell death induced by NSC777201 in CRC cells. Alternatively, NSC777201 induces autophagy and ferroptosis in CRC cells. Because malignant cells usually develop apoptosis resistance, resulting in drug resistance and clinical treatment failures, the ability of NSC777201 to trigger multiple cell death pathways may provide new therapeutic opportunities.

The tumor suppressor gene, *TP53*, is mutated in more than 60% of CRC cases. Functional p53 is usually required to induce apoptosis in response to DNA damage induced by chemotherapeutic agents [36]. Thus, *TP53* mutations may confer resistance to systemic treatments, which can profoundly impact patient treatment responses and outcomes [37]. According to the NCI-60 screening and our results, we found that NSC777201 significantly exhibited tumor inhibitory effects in different CRC cells harboring either the wild-type (HCT116, KM12, HCC2998, and RKO) or mutant *TP53* gene (HT29, SW620, COLO205, HCT15, and DLD-1). Therefore, the anticancer activity of NSC777201 appears to be independent of the *TP53* status, providing a novel strategy for treating CRC.

Cholesterol is closely related to cell death, including autophagy and ferroptosis. In general, cholesterol plays an inhibitory role in cell death. For example, cholesterol depletion promotes autophagosome formation [38]. In addition, cholesterol was shown to reduce membrane fluidity and promote lipid raft formation, thereby inhibiting lipid peroxidation and ferroptosis in cancer cells [39]. Based on the RNA-Seq analysis, NSC777201 may reduce cholesterol levels in CRC cells, leading to the compensatory upregulation of genes related to cholesterol biosynthesis. This hypothesis warrants further investigation.

## Conclusions

NSC777201 induces CRC cell death by triggering autophagy, ferroptosis, and ferritinophagy. Mechanically, NSC777201 inhibits GPX4, thereby increasing lipid peroxidation and leading to ferroptosis. Additionally, NSC777201 inhibits FTL, releasing ferric ions to further induce ferroptosis. Moreover, NSC777201-induced ferroptosis is partly mediated by the upregulation of DHCR7 and a reduction of endogenous 7-DHC levels. Our findings demonstrated that NSC777201 can induce multiple types of cell death and may serve as a promising therapeutic agent for CRC in the future.

## Data Availability

The datasets used and analyzed during the current study are available from the corresponding author on reasonable request.
